# Making sense of expanding transcriptomic data: network-based approaches for studying reproduction in domestic and wild animal species

**DOI:** 10.3389/fvets.2025.1728981

**Published:** 2026-01-12

**Authors:** Olga Amelkina, Pierre Comizzoli

**Affiliations:** 1 Department of Reproduction Biology, Leibniz Institute for Zoo and Wildlife Research, Berlin, Germany; 2 Smithsonian's National Zoo and Conservation Biology Institute, Washington, DC, United States

**Keywords:** machine learning, network visualization, non-model animal species, pathway enrichment, reproduction, transcriptomics

## Abstract

Transcriptomic datasets in animal reproductive biology are expanding rapidly, creating more opportunities to explore genome-phenome relationships, uncover biological mechanisms, and improve assisted reproductive technologies. This mini-review emphasizes the shift from single-gene analyses to a systems biology approach, where genes and pathways are studied within networks to capture their interactions and better understand biological systems. We show how network visualization can help synthesize knowledge from complex RNA-seq outputs and provide examples of tools and workflows suitable for species with different levels of data availability and annotation. Best practices for data generation and integration from various databases are discussed, highlighting the importance of high quality well-annotated datasets, transparent reporting, and the pitfalls of overinterpretation. Machine learning methods are explored as an analysis option for experiments with hundreds of data points. Ultimately, expanding available expression datasets for non-model species, combined with rigorous data processing and interpretation, will enable reproductive biologists to integrate network-based strategies into their research and advance reproductive science as well as conservation programs.

## 1 Introduction

High-throughput sequencing technologies are becoming affordable and accessible, resulting in an unprecedented rate of data accumulation, especially from transcriptomics studies. These datasets can help researchers investigate cellular mechanisms, uncover novel biological functions, identify associated molecular markers, and explore genome-phenome relationships (1–3). However, the analysis and interpretation of such complex, multidimensional data, defined by numerous interconnected variables, remain a major challenge, particularly in fields outside of cancer research where machine learning (ML) methods and network-based approaches have already been widely adopted (4, 5).

Genomics and systems biology share a common ground in understanding biological processes through integrative analysis, using experimental and computational methods (6). In essence, systems biology aims to study biological systems by first perturbing them, then measuring resulting gene, protein, or pathway responses, integrating the observed data, and ultimately modeling these data to describe the structure of the system and its response to perturbations (7). Tools such as pathway enrichment, gene regulatory networks, and knowledge graphs offer powerful means to visualize data structure and interpret it within the broader context of biological function, while ML methods such as artificial neural networks can add predictive power (the ability to predict outcomes) and identify key drivers of observed transcriptional changes. Introducing these strategies more broadly into the field of animal reproduction biology can advance understanding of reproductive mechanisms and accelerate the development of assisted reproductive technologies (ARTs) in domestic and wildlife species. Our recent studies demonstrate the use of network visualization in interpreting transcriptomic data to study gonadal tissue development and response to preservation protocols in the domestic cat (8–10), and combination of transcriptomic and proteomic data to analyze semen composition and environmental response in the endangered black-footed ferret (*Mustela nigripes*) (11), highlighting the potential of this approach in fields of biobanking and conservation. Meanwhile, mice and cattle benefit from extensive annotation, vast tissue expression databases and high sample size to leverage ML methods to generate novel reproductive insights and fertility predictions (12–16).

The objective of this mini-review is to introduce network-based strategies for interrogating and integrating transcriptomic data to community of reproductive biologists, as well as advice on best practices and potential pitfalls. We provide an overview of databases, visualization tools and workflows suitable for model and non-model animal species, with a primary focus on bulk RNA sequencing (RNA-seq) studies. References to comprehensive reviews and analysis pipelines are included throughout, and readers are encouraged to consult these sources prior to undertaking large-scale analyses. While this review focuses on transcriptome, introduced strategies and tools can also be used when working with other types of omics data, such as genome, epigenome, proteome, metabolome, and lipidome. Ultimately, integrating various layers of data makes the research even more powerful (17, 18).

## 2 Transcriptomic data layers in reproduction

Transcriptomic data generated from RNA-seq provides a dynamic snapshot of gene activity and gives the ground for exploring various reproductive processes (19–21). Before introducing network-based analysis tools, it is essential to consider what each transcriptomic layer can reveal about the biological system and to outline practical strategies for generating high-quality data or sourcing relevant datasets from public repositories.

### 2.1 Coding transcriptome

The coding transcriptome represents mRNAs that are ultimately translated into proteins and drive cellular function. While bulk RNA-seq is used widely to profile gene expression across reproductive tissues and developmental stage, single cell (sc) and spatial RNA-seq (22) provides cell-level resolution and allows to dissect heterogeneity of reproductive tissues (21).

#### 2.1.1 Generating your own data

Careful experimental design is critical when generating your own transcriptomic data. Biological replication is one of the key considerations and should reflect expected variability in the population (23). Todd et al. provide an excellent guide for organizing your RNA-seq experiment in non-model species, connecting sample size, statistical power and effect size for various degrees of genetic variation (24). Sequencing depth is another important consideration and a good rule of thumb for mammalian transcriptome would be 150 paired-end sequencing with 30 million read depth per each biological replicate/library to get a comprehensive transcriptome data, while 10 million read depth may be enough for simple differential expression comparison of highly expressed genes (23, 25). Finally, RNA quality itself must be good enough as low-quality RNA results in uneven gene coverage, higher false-positive rates during differential expression analysis, higher duplication rates and negative correlation with library complexity (26, 27). For mammalian RNA, solutions to stabilize RNA such as RNAlater can help with preserving RNA integrity, especially during field collections (28). If there is no possibility of obtaining a good quality RNA, there are library preparation methods that were adapted to reduce the effect of RNA degradation (29, 30), as well as bioinformatic tools to account for bias toward shorter RNA species (31). Well-annotated reference genomes boost accurate alignment and interpretation (32), but if they are not available for particular wild species, comparing results of de-novo transcriptome assembly and alignment to the related species genome can help with initial investigation (33, 34).

#### 2.1.2 Utilizing publicly available datasets

Public repositories such as GEO (35) and SRA (36) host numerous RNA-seq datasets for reproductive tissues. For model species, there are also species-specific resources available, such as gene expression database GXD (37) and recount3 (38) for mouse, and cattle genotype-tissue expression atlas CattleGTEx (39). For non-model species, data availability is more limited and often requires searching individual publications, highlighting that comprehensive expression atlases and so called digital biobanks (40) for at least domestic models for wildlife species (such as domestic cat) are critically needed.

When reusing public data, good processing step is essential. To minimize batch effects and annotation drifts, raw reads can be re-quantified using a consistent pipeline and current reference genome. Converting raw reads to Transcripts Per Million (TPM) normalizes relative molar concentration of transcripts per sample and it is a baseline quantification recommended when working with RNA-seq (41), unless the method specifically uses raw reads (42). When integrating datasets from various sources, it’s important to apply metadata standardization, such as tissue type, developmental stage, sequencing parameters, as well as batch correction methods, for example ComBat-seq (43) and svaseq (44).

### 2.2 Non-coding transcriptome

Beyond coding mRNAs, non-coding RNAs add critical regulatory layers in reproductive processes. Among these, microRNAs (miRNAs), long non-coding (lnc) RNAs, and resulting competing endogenous (ce) RNAs are particularly relevant for network-based analyses.

#### 2.2.1 MicroRNAs

miRNAs regulate gene expression primarily through mRNA degradation or translational inhibition (45). While degradation effects can be inferred from measured mRNA levels, translational inhibition often requires proteomic data for confirmation. Although miRNAs are highly tissue- and stage-specific (46), their high sequence and target conservation status across species helps with miRNA identification in non-model animals by utilizing orthologs of well-studied species (47).

While miRBase database is the most commonly used for identifying miRNAs (48), it is limited in its incomplete species coverage and naming inconsistencies. Another database MirGeneDB is hand curated and extremely reliable (49), however, the miRNA names from this database are rarely used in publications and chemical company catalogs. Tools like miRDeep that identify known and novel miRNAs, as well as orthologs based on the databases provided, are very useful in miRNA studies for both domestic and wild animal species (50). Separately, for mouse a whole miRNA tissue expression atlas (miRNATissueAtlas) is already available (51). Validated and predicted targets of miRNAs can either be sources from existing databases such as miRTarBase (52), miRDB (53), TargetScan (54) and miRWalk (55), or predicted for specific species based on uploaded 3’UTR sequences (54). miRmapper is a useful R package that allows to collect targets from most of the available databases (56).

#### 2.2.2 Competing endogenous (ce) RNAs

The ceRNA hypothesis, first proposed by Salmena et al. in 2011 (57), describes how lncRNAs, circular (circ) RNAs and pseudogenes compete for shared miRNAs, indirectly regulating each other’s expression. lncRNAs can act as miRNA sponges to prevent them from binding to their target mRNA, while circRNAs influence transcriptional and post-transcriptional regulation by interacting with spliceosomal components (58).

Databases for lncRNAs and circRNAs exist primarily for model species (59), and low sequence conservation limits cross-species applicability. However, computational prediction of miRNA binding sites on lncRNAs and circRNAs allows to construct ceRNA network in non-model species (60, 61).

## 3 Network-based approaches for biological interpretation

Network-based approaches provide a powerful tool for interpreting complex transcriptomic data by representing biological systems as interconnected entities (62). In these models, nodes usually correspond to genes, proteins, or pathways, while edges represent relationships between the nodes such as co-expression, physical or functional interaction, or regulation. Edges can be undirected, e.g., correlation based, or directed, indicating causal interactions with a sign (activation or inhibition) and context (phosphorylation, transcriptional activation, repression) (63). Visualization tools such as Cytoscape allow one to build their own networks, explore them with various layouts and add additional data layers, integrating topology with functional annotations (64, 65).

### 3.1 Pathway enrichment and visualization

RNA-seq experiments often produce extensive lists of differentially expressed genes (DEGs), which are difficult to interpret through manual literature review alone. Functional enrichment analysis provides a systematic approach by identifying statistically overrepresented biological pathways and function (66, 67). However, enrichment outputs can themselves be overwhelming, often comprising long, redundant lists of terms. Network visualization mitigates this by clustering related gene sets into coherent themes, reducing redundancy and helping to grasp the overall picture of enrichment results.

**Figure 1:**
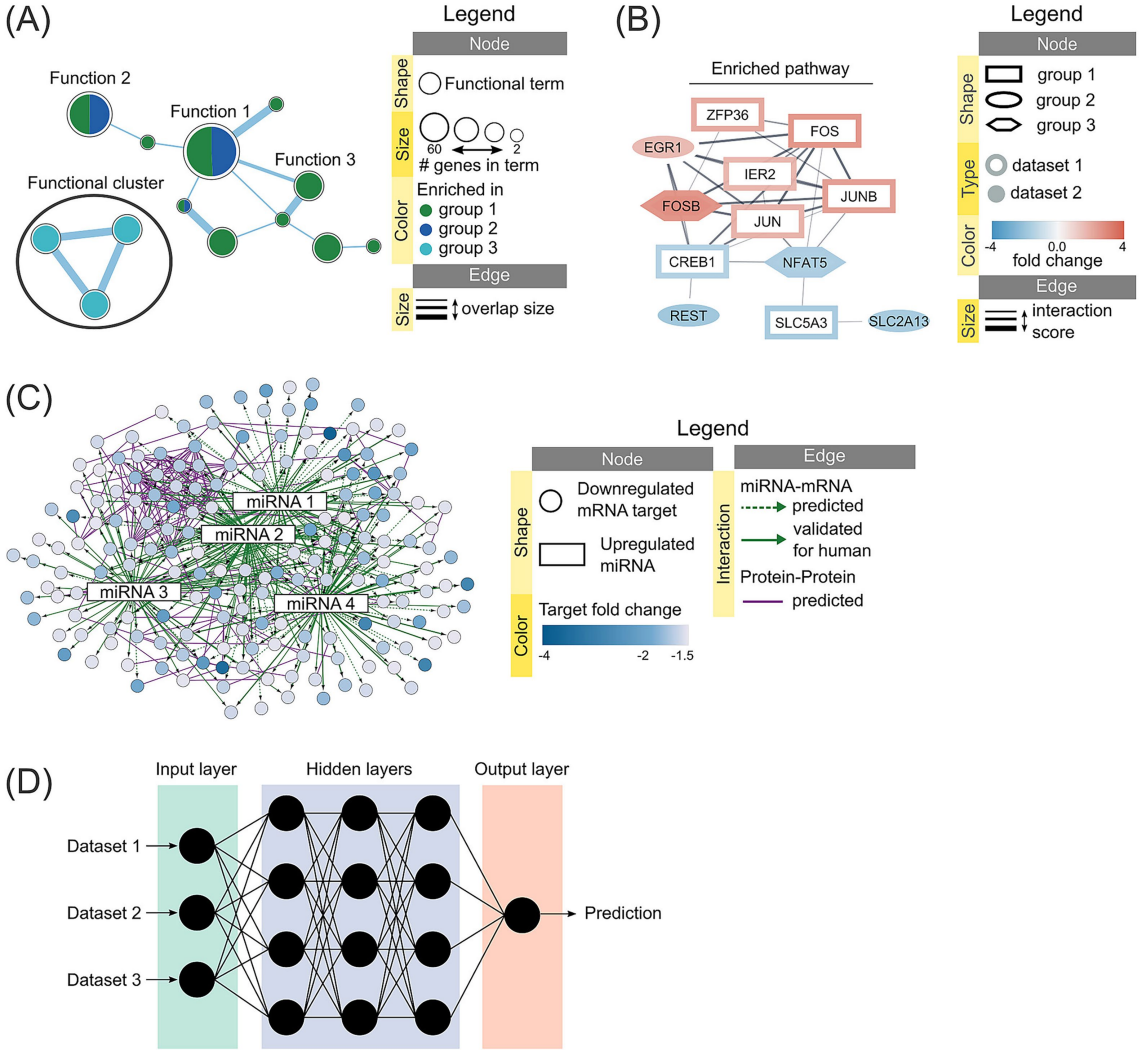
Examples of several types of biological networks and their legends. **(A)** Network generated with EnrichmentMap in Cytoscape representing various pathways and functions enriched in the analyzed groups. The legend describes nodes and edges (connections between the nodes) which can be annotated with colors and different sizes. Here node size corresponds to the number of differentially expressed genes (DEGs) enriched in the term, color corresponds to the experiment group where the term is enriched in, while edge thickness shows how many genes are shared between the terms (thicker edge means more genes). **(B)** Protein–protein interaction (PPI) network generated with stringApp in Cytoscape representing DEGs and their predicted or validated interactions. Node shape represents the experiment group, border/body coloring represents the DEG dataset, color represents fold change for each DEG, while edge corresponds to the interaction score from STRING database (thicker edge means more evidence for this interaction). **(C)** miRNA-mRNA-PPI network generated in Cytoscape using prepared table of miRNA-mRNA interactions and PPI data from stringApp. Edges are annotated to represent different interaction types, including miRNA-mRNA and PPI. **(D)** Neural network representing deep learning approaches of machine learning, where the number of hidden layers defines the depth of the network. Processed data is fed into the input later and is then transformed inside the hidden layer into a representation that is learned and fed forward to the next layer. Model gets tuned for higher performance by backpropagating errors made on the training data. Based on the tuned hidden layers, the output layer generates a prediction, which can be either classification or regression type.

EnrichmentMap (68), available as a Cytoscape app (69) and also as a web tool (70), is a tool of choice for this. It organizes enrichment results into networks where nodes are enriched terms and edges represent gene overlap between the terms, see example in Figure 1A. Additional Cytoscape apps such as clusterMaker (71), WordCloud (72), and AutoAnnotate (73) further enhance interpretability by grouping and labeling clusters. This protocol (74) provides a great example of enrichment analysis pipeline with EnrichmentMap visualization.

The choice of enrichment tool depends on species coverage and the statistical approach, i.e., predefined gene set vs. ranked gene list from differential expression analysis. For instance, DAVID (75) performs gene set enrichment and supports annotations for over 50 species, including domestic cat and ferret, making it a good choice for non-model species analysis. Meanwhile, GSEA (66) operates on ranked gene list but is limited to human and mouse annotations, relying on the MSigDB database; therefore, analysis in other species require orthology mapping. Finally, g: Profiler (76) supports over 800 species via Ensembl and allows custom annotations, making it another great choice for non-model species analysis. Outputs from all three of the above enrichment tools can be then visualized with EnrichmentMap. Ideally, species-specific databases should be used to avoid missing lineage-specific genes. On the other hand, human and mouse annotation databases provide more functional information and can be used to expand the results and generate additional hypotheses. However, this should be done carefully and only in addition to the species-specific annotation to not over-interpret your results: some species-specific genes, particularly those involved in immune and reproductive functions, may lack direct counterparts in human or mouse, limiting transferability. For a broader overview of gene set enrichment analysis see (77).

Beyond standard enrichment analysis, topology-aware methods consider the actual structure and direction of pathways. For example SPIA (78) combines classical enrichment with the analysis of how a pathway is perturbed under specific conditions, and it is available for numerous species annotated in KEGG database, including domestic cat and ferret. Other approaches, such as PROGENy (79), estimate pathway activity based on downstream gene responses, while CARNIVAL (80) reconstructs causal networks by linking interactions that have both direction and sign. These methods are particularly useful for studying signaling pathways, but are often limited to human and mouse, requiring orthology mapping for other species.

### 3.2 Protein–protein interaction (PPI) networks

Unlike traditional approaches that focus on individual DEGs, network-based strategies capture gene interactions, which have been shown to be more predictive of phenotype than single-gene markers (81). PPI networks position DEGs within interaction maps, revealing functional clusters and signaling cascades, and including such analysis on top of the initial pathway enrichment can add confidence in the observed data trends. STRING database integrates experimental and predictive evidence for both functional and physical interactions of proteins for many species (82, 83), and StringApp in Cytoscape (84) allows to map additional information to the network, see example in Figure 1B. Mice studies can utilize GeneMANIA (85) and it’s Cytoscape app (86) which collects numerous interactions from various databases, and expand it by addition of transcription factor binding information from manually curated TRRUST v2 database (87). For cattle, domestic cat and ferret, AnimalTFDB 4.0 database (88) can be used for predicted transcription factors.

When PPI networks are too large they resemble so called “hairballs” and require the use of clustering methods to improve network readability (89). Cytoscape apps such as clusterMaker (71) and MCODE (90) can extract PPI clusters based on their interaction score, which can then be analyzed for functional enrichment (e.g., integrated function in StringApp) to improve biological interpretability. Utilizing human orthologs, CORUM database (91) can also be used to extract known mammalian protein complexes.

### 3.3 Gene regulatory networks (GRNs) and integration strategies

GRNs can model relationships between regulators (transcription factors, miRNA) and targets (mRNA) and are a powerful abstraction of biological systems (62, 63); see example in Figure 1C. Zhao et al. (92) provide an in-depth overview of tools for inferring GRNs from various types of expression data, dividing them into model-based, information-based and machine learning-based methods. Model-based methods, including differential equation, Boolean and Bayesian methods, are suitable for inferring small real networks, while information theory-based methods, including Pearson correlation coefficient and (conditional/part) mutual information, are suitable for steady-state data (92). Machine learning-based methods, such as widely used tool GENIE3 that utilizes random forests to infer GRN (93) and its adaptation for time-series expression data dynGENIE3 (94), provide best results when reconstructing large-scale networks. In addition, reverse engineering approaches have been developed to trace back the initial relationships between genes that resulted in the observed gene expression (62). Mercatelli et al. (95) go through a variety of tools available for GRN inference, and highlight that the optimal tool selection ultimately relies on the biological context studied and data availability on the species, transcription factors, cellular context or specific perturbation.

Integrating strategies can extend GRNs to multi-layer networks by incorporating miRNA-mRNA interactions, ceRNA relationships, genomic variants and other omics data layers. Morabito et al. (96) provide a useful overview of recent algorithms and tools applied to integrate genomics, transcriptomics, proteomics, and metabolomics, while a more ML oriented approach can be found in the review of Picard et al. (97).

### 3.4 Machine learning and neural networks

Machine learning uses computation to recognize patterns in the data by fitting predictive models to it or identifying informative clustering within data (98). This approach allows scientists to make predictions where experimental data is lacking to guide future research and to improve the understanding of biological systems. Greener et al. (98) provide an essential guide to ML for biologists that is a good starting point, which can further be expanded with practical advices from Chicco (99) and more in-depth review of use for biological networks from Camacho et al. (100).

ML methods are divided into supervised, where a model is fitted to labeled data, and unsupervised, where a model identifies patterns in unlabeled data (98). Apart from traditional ML, an area of deep learning that relies on neural networks has been rapidly developing for genomics analyses (101, 102), see example in Figure 1D. While deep learning is a powerful tool, it is limited to specific applications where a large amount of highly structured data is available, i.e., each data point has many features with clear relationship (103). Generally, traditional ML should be the starting point to find the most appropriate method for a given analysis and in some cases outperforms deep representation learning in phenotype prediction from transcriptomics data (81). At the same time, interpretable ML for omics data is becoming more prevalent in systems biology (104).

Because ML typically requires large amount of data points, from hundreds and thousands for traditional ML to millions for deep learning, as well as training and validation sets for supervised methods, this approach may not always be applicable to bulk transcriptomic data in non-model species. When sample size is limited, applying unsupervised ML methods, such as t-SNE (105) and UMAP (106), to scRNA-seq data can enable detailed profiling of reproductive tissues and their cellular environment, providing foundation for *in vitro* system development and generation of further hypotheses. Here single-nuclei RNA-seq approaches are particularly useful in field conditions, as tissues can be snap-frozen in liquid nitrogen without immediate dissociation (107). In addition, genomics data is growing fast for wild species and opens possibilities to study evolutionary trends in reproduction with the use of ML in the future, including miRNA emergence in placental animals (108) and effect of deleterious mutations on species fitness (109, 110).

## 4 Limitations when working with omics data

**Figure 2:**
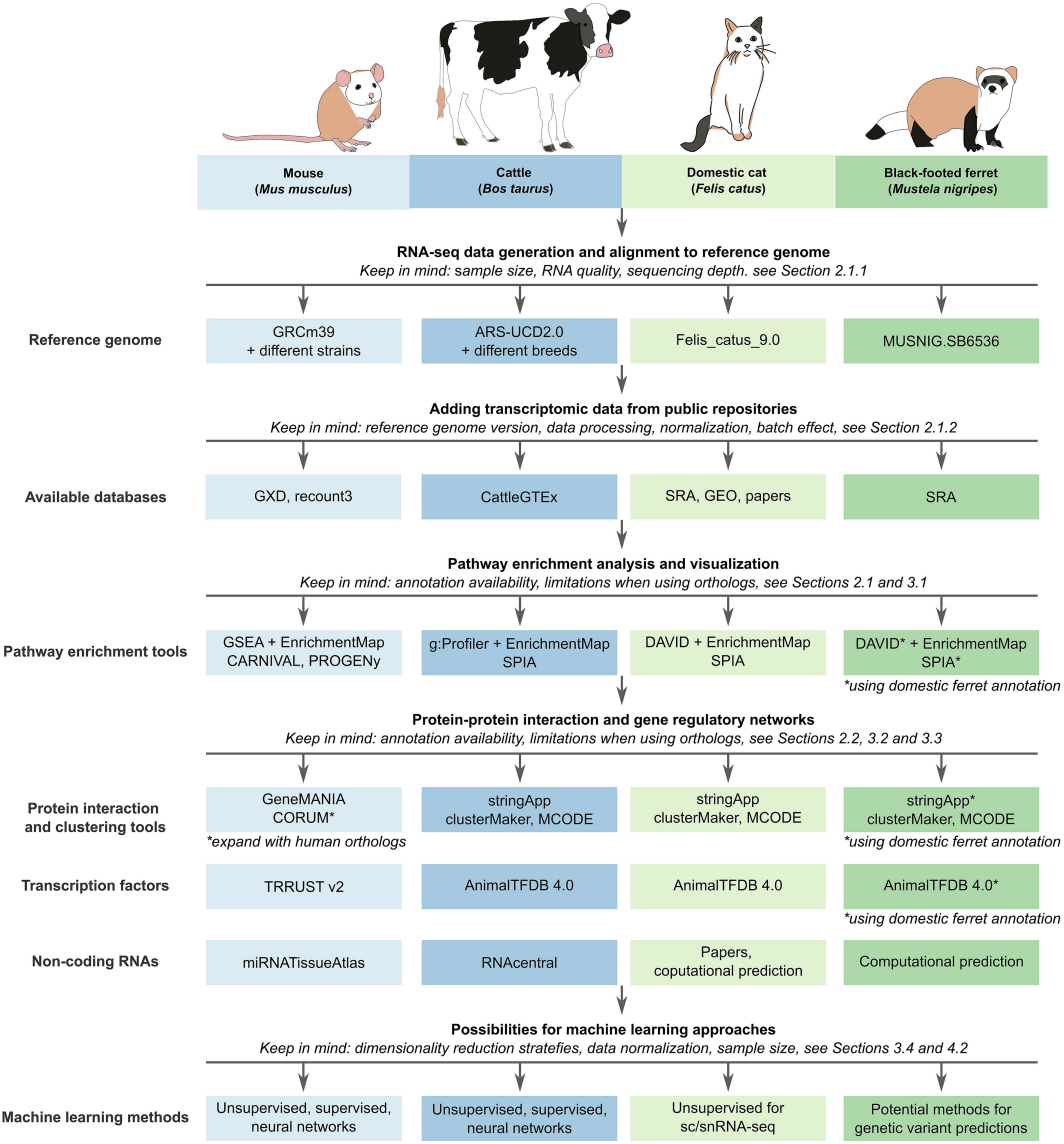
Comparison of workflows for species with different levels of annotation and prior knowledge. A model species like the mouse and a domestic species like cattle both benefit from a high number of available expression datasets and experimental knowledge, allowing the use of supervised machine learning methods. A less studied species like the domestic cat has a limited availability of annotated databases and often relies on prediction algorithms or orthology mapping. A wild species like the endangered black-footed ferret must rely on annotations from the domestic ferret but can utilize genomic information for further studies.

Despite the promise of transcriptomics, ML and network-based approaches, several challenges constrain their interpretability and predictive power. Figure 2 illustrates how workflows differ between a well-studied species (mouse and cattle) and non-model species (domestic cat and black-footed ferret), highlighting the impact of database availability.

### 4.1 “Garbage in, garbage out”: the incentive for high-quality data

The principle of “garbage in, garbage out” is borrowed from computer science and can be applied directly to omics analysis: flawed input data inevitably produce misleading predictions and interpretations. For transcriptomics-based phenotype prediction, proper normalization and robust regression methods are essential, but the factors that have the biggest impact still consist of adequate biological replications and sequencing depth, ensuring accurate sample metadata, incorporating complementary data types such as proteomics, and improved prior knowledge. Paton et al. demonstrated that preprocessing choices significantly affect downstream enrichment results (111). In order to trust the results of network inference and be able to apply ML methods in a study, researchers must produce/utilize high-quality raw data and provide transparent reporting of quality control and processing.

### 4.2 The “curse of dimensionality” and strategies to overcome it

Termed as the “curse of dimensionality,” a common problem in omics datasets is that the number of features is much higher than the number of samples. This leads to overfitting and poor generalization in ML models (104). Dimensionality reduction through feature extraction, selection or engineering can reduce the number of variables (data sparsity) and improve prediction reliability (112). Lasso regularized models and nearest shrunken centroids introduce sparsity by removing contributions of unimportant features (113), as used in the example of combining transcriptomics and genetic variants to predict phenotype from genotype (114).

### 4.3 Overinterpretation problem

While omics data and its integration into network-based or ML approaches are powerful methods to infer observed changes in biological systems, they can also lead to overinterpretation when patterns are mistaken for causal relationships. Hub nodes, for example, often reflect annotation density rather than true biological centrality. Similarly, inferred regulatory relationships of nodes via edges should be treated as hypotheses rather than definitive interactions, particularly in non-model species where prior knowledge is sparse. It is always best practice to report data quality and confidence scores, be careful when discussing results and, when possible, validate predictions experimentally. Ultimately, insight gained from computational analysis and network visualization should rather guide hypothesis formulation and future functional studies but never replace them.

## 5 Future directions

Future progress in reproductive biology depends on closer integration with the genomics community to connect fertility and reproductive adaptations to genomic variation. While genome-to-phenome mapping and fertility prediction are rapidly advancing in model species and livestock through genome/transcriptome-wide association studies and (e)QTL approaches, non-model species and wildlife remain behind, with genomic data rarely linked to reproductive phenotypes. Emerging genomics studies are applying genetic load estimates to guide breeding strategies and reduce inbreeding depression in captive populations (115). Integrating fertility and expression data into such approaches will enhance their efficiency and strengthen conservation breeding efforts. Network-based approaches provide a natural framework for this integration.

To achieve this, we will need more reproductive expression datasets and species-specific digital biobanks where genomics meets transcriptomics, proteomics, and ultimately fertility phenomics – “fertilomics.” These resources, combined with multi-omics integration, advanced network visualization tools, and ML, will enable predictive modeling and comparative analyses across evolutionary contexts.

## 6 Conclusion

Network-based approaches provide a powerful framework for interpreting transcriptomic data, enabling integration of complex datasets, hypothesis generation, and guiding functional studies. Adopting the strategies discussed in this mini-review would drive the transition from single-gene interpretations to systems-level approaches in reproductive studies. Expanding available expression datasets for non-model species, combined with best practices in data quality, processing and careful interpretation of results will allow the reproductive biology community to efficiently integrate network-based and ML strategies into their research and advance evolutionary and reproduction research, ARTs and conservation programs.
